# Economies of scale of large-scale international development interventions: Evidence from self-help groups in India

**DOI:** 10.1016/j.worlddev.2022.105839

**Published:** 2022-05

**Authors:** Garima Siwach, Sohini Paul, Thomas de Hoop

**Affiliations:** a Senior Economist, American Institutes for Research, 4700 Mueller Blvd., Austin, TX 78749, United States; b Senior Program Officer, Population Council, United States; c Principal Economist, American Institutes for Research, United States

**Keywords:** Costing, Scaling up, Cost-effectiveness, Self-help groups, Economies of scale, India

## Abstract

Livelihoods and microfinance programs for women often show reduced impacts after scale-up. Yet, program scale-up may reduce average per capita costs and maintain cost-effectiveness despite lower impact. This paper presents evidence on the association between program scale, costs, and cost-effectiveness by analyzing how the costs of a large-scale Self-Help Group (SHG) program in India changed from its inception in 2007 to its scale-up in 2019. We use expenditure data from program’s audit statements of *Jeevika* – the Bihar Rural Livelihoods Promotion Society – and find that a 1% increase in program membership was associated with a 0.6% increase in annual program expenditures, indicating large economies of scale. Predicted costs from regressions suggest that the annual per capita program expenditures declined from $29 when the program covered 100,000 members to $5 when it reached 10 million members. Previous impact evaluations of *Jeevika* showed sizeable but smaller substitutions away from high-cost debt after scale-up than during the pilot, but we found that economies of scale led to similar cost-effectiveness ratios for this outcome. We also found that formation of higher-level federations is associated with lower marginal costs than setting up SHGs. However, previous evidence suggests that *Jeevika* did not generate average impacts on women’s agency and asset ownership after scale-up. Building on a rich history of research on *Jeevika*, we argue that program implementers must identify key success factors in pilot programs to minimize tradeoffs between cost savings and potentially reduced impacts after scale-up. Further, we suggest investments in linking SHGs to federations to improve the cost-effectiveness of SHGs.

## Highlights

• Large scale international development interventions can realize significant cost savings due to economies of scale.
• 1% increase in program membership in an SHG program in India was associated with 0.6% increase in annual program expenditure.
• The program had similar cost-effectiveness for primary outcome at scale as in the pilot; pther impacts decreased at scale.
• Formation of SHG federations, specifically, can gain from large cost efficiencies when implemented at scale.
• Programs must identify key success factors to minimize tradeoff between cost savings and implementation quality at scale.

## 1 Introduction

In recent years, the developing world has seen an increased focus on institutionalizing women’s groups with economic objectives as a key channel to improve women’s empowerment and economic outcomes. These interventions include self-help groups (SHGs), savings groups, mothers’ groups, health groups and community mobilization groups (Dìaz-Martin et al., 2020). While interventions that involve women’s groups have a long history in community development, these networks were traditionally more informal and disaggregated than they are today. Many countries are moving towards large-scale investments to mobilize women in groups and use these groups as delivery vehicles for various economic and health initiatives. India’s National Rural Livelihoods Mission (NRLM) has mobilized over 70 million households into SHGs with the goal of providing them with access to financial services and sustainable livelihoods enhancements (Ministry of Rural Development, 2011). The Nigeria for Women Project also recently began mobilizing 0.32 million women into Women’s Affinity Groups to introduce them to group-based savings, credit, and livelihoods programs (World Bank, 2018a).

To some extent, these decisions to scale are justified by evidence on the impact of women’s groups. A systematic review showed that SHGs have positive effects on several domains of women’s empowerment—including economic, reproductive, social, and political domains—although impacts depend substantially on program design and implementation context (Brody et al., 2015). Apart from SHGs, research also shows that open, community groups of women that involve participatory learning can improve maternal and newborn health in low-resource settings (Prost et al., 2013). Some studies attribute these greater benefits to accumulation of social capital and mutual accountability resulting from peer interactions and social exchanges facilitated by groups (Gram et al., 2019, Brody et al., 2015). However, a recent evidence synthesis by Dìaz-Martin et al. (2020) suggests that the benefits of women’s group programs primarily result from leveraging groups as a platform to deliver programming to many women at once, suggesting that groups may be able to deliver benefits at a lower cost per program participant. Based on this finding and the limited evidence on the costs and cost-effectiveness of women’s groups with economic objectives, the authors argue that more research is needed to understand the overall costs of group models, including costs of creating and mobilizing groups, and the operational costs of group-based activities (Dìaz-Martin et al., 2020).

Given the growing interest in expanding the outreach of women’s groups with economic objectives, it is imperative to study the potential role of scale in influencing program impact, implementation, and costs of these groups. Without such information, the policy implications of studies examining the impact of women’s groups operating at scale will remain unclear. While various studies examine the costs and cost-effectiveness of women’s groups aiming to improve health outcomes (Mangham-Jefferies et al., 2014, Colbourn et al., 2015, Sinha et al., 2017, Pulkki- Brännström et al., 2020), somewhat paradoxically there is limited information on the costs of women’s groups with economic objectives.

This paper addresses this evidence-gap by examining how the costs and cost-effectiveness of an SHG program in India changed with respect to program scale. We study how the costs of a large-scale SHG program in the state of Bihar – *Jeevika* – changed over time since its inception in 2007 when it mobilized 8,000 women into SHGs, to its current reach where it has mobilized over 11 million women into 0.9 million SHGs. *Jeevika* has operated the Bihar Rural Livelihoods Project (BRLP) since 2007, and currently implements India’s NRLM through Bihar’s State Rural Livelihoods Mission (SRLM). The NRLM, which was launched in 2011, operates in 28 states through the SRLMs, which create and work with women’s SHGs to facilitate institutional and capacity building, financial inclusion, livelihoods promotion, social inclusion, and development. SHGs under NRLM (and BRLP) usually start with a period of collective savings to facilitate intragroup lending, after which members can gradually take larger loans (usually from formal financial institutions like banks). Additionally, to facilitate collective capacity, *Jeevika* created a federation of community institutions, under which SHGs were linked to Village Organizations (VOs) and VOs were linked to Cluster-Level Federations (CLFs). Among other activities, these federations provide training to improve agricultural and non-agricultural livelihoods and health outcomes, including training for some of the most disadvantaged groups.

Program scale may impact outcomes through different mechanisms. On the one hand, increases in scale may lead to an adverse impact on implementation quality leading to lower effectiveness. A recent *meta*-analysis of development programs, including those that focus on microfinance, financial literacy and women’s empowerment, concluded that larger programs and programs that were implemented by governments had lower effects (Vivalt, 2020). Indeed, program-specific evidence on other development interventions suggests that many pilot programs that are successful at a smaller scale fail to achieve similar positive effects after scaling up (Bold et al., 2018, Bryan et al., 2014). More recent evidence indicates that prior experience in communities could explain the larger positive effects of interventions implemented by non-governmental organizations (NGOs) relative to interventions implemented by governments (Jeuland et al., 2020).

On the other hand, service scale may also alter the costs of service provision if programs are able to achieve economies of scale (Tulloch, 2019). Previous evidence from health programs and interventions aiming to reduce gender-based violence indeed show that the average costs of these programs reduce significantly when they reach a larger number of program participants (Abramsky et al., 2012, Bijlmakers et al., 2018, Brooker et al., 2008, Jan et al., 2011). At the same time, more complex interventions like multidimensional livelihoods and financial inclusion interventions, may achieve diseconomies of scale if the programs face input or labor supply constraints (Brown & Tanner, 2019). The scale-up of SHG programs and other development interventions could either increase or decrease their cost-effectiveness depending on how scale changes the implementation quality, effectiveness, and costs of programming. In the absence of cost information, and specifically cost information about programs delivered at different levels of scale, the returns on investment of large-scale programs as well as the policy implications of the studies showing an adverse relationship between impact and scale remain unclear.

Implementers must also identify elements that are imperative for the cost-effective implementation of women’s groups programming at scale. Identifying these elements requires assessing the marginal costs of adding different program activities. Prior costing analyses and systematic reviews of SHG programs point to a trade-off between cost savings and benefits from increasing the scope of services. Evidence suggests that both benefits and costs of SHGs increase when SHGs deliver an increased number of activities, such as trainings focused on empowerment, social change, and livelihood generation (e.g., Brody et al., 2015, Isern et al., 2007, Tankha, 2002, Harper, 2002). However, studies presenting cost-effectiveness analyses of women’s groups with economic objectives (Deininger and Liu, 2013, Karlan et al., 2017) have not yet examined the costs of specific program components or the contextual determinants of these costs. In the absence of reliable cost data, it thus remains unclear whether adding in extra programming to group-based models with economic objectives will increase their cost-effectiveness, though some evidence indicates that group-based models can indeed achieve the same outcomes at a lower cost than programs targeting individuals (Grantham-McGregor et al. 2020).

Using program expenditure data from annual audit reports of *Jeevika* between 2007–08 and 2018–19, we contribute to the literature by estimating costs of different program components, including capacity building, institutional development, and project management. We use these estimated costs to empirically test for evidence of economies of scale. In addition, we provide evidence on how costs of the *Jeevika* program change with the inclusion of extra programming, and how program cost-effectiveness may change with scale. In this way, we respond to the need for more research on the costs of group models and the operational costs of group-based activities identified by Dìaz-Martin et al. (2020).

Understanding how costs change with scale is challenging since the nature of program and the number of activities often change as programs expand. Studies have pointed out that a good way to differentiate between short-run and long-run costs is by exploiting time-series data on program costs – a challenging endeavor given the lack of data on costs over time (Johns & Torres, 2005). While a large dataset with data on costs of multiple programs over many periods and program components would be ideal, such data is almost never readily available. Instead, most studies observe cost data at one point in time or have limited data on the costs of different program components precluding researchers from studying how costs vary with changes in both program scale and program scope. Therefore, even a small increase in the number of cost data points over time can significantly increase the evidence-base on how costs change with scale, especially when data include costs of different program components. Time-series data are particularly important for women’s groups like SHGs, that usually start with the goal of increased financial access to women, but with time, often include additional layers like health or farm and non-farm-based livelihoods initiatives.

So far, researchers have examined how the effectiveness and implementation of SHGs and specifically the *Jeevika* program varies with scale of programming (Hoffman et al., 2018, Majumdar et al., 2017). Findings from impact and process evaluations of *Jeevika* suggest that the impacts of the program on women’s empowerment and economic outcomes reduced after expansion in the pace and scale of implementation because of a lighter program touch (Hoffman et al., 2018). Evidence from other programs suggests that scaled-up programs may have larger general equilibrium effects through changes in prices and wages (Breza and Kinnan, 2018, Muralidharan et al., 2020). The evaluation of the scaled-up *Jeevika* program suggests that the program led to increased access to formal credit, and reduced interest rates charged by informal money lenders (Hoffman et al., 2018, Kochar et al., 2020). The reductions in interest rates resulted in particularly large benefits for marginalized populations, such as scheduled caste (SC) and scheduled tribe (ST) households and landless households. The program reduced the difference in average borrowing rates for landholding and landless households by two thirds, and had positive effects on ownership of productive and consumption assets for landless households (Hoffman et al., 2018).

*Jeevika* makes a compelling case for examining how scale influences program costs. The program originally started in six priority districts of Bihar with funding from the World Bank in 2006–07, before NRLM was formally launched by the Government of India. In 2011, with additional funding from the World Bank as well as the launch of NRLM, *Jeevika* scaled up its operations beyond the originally planned six districts. In our data, we observe program expenditures over the short-run when the program operated at smaller scale, and in the longer-run when the program scaled up across the entire state. While the dataset remains relatively small, the time-series cost data enable us to distinguish between short-run and long-term costs during the pilot and after the scale-up of the program. The period of 12 years for which we obtained cost data is typical for long-term scaling-up processes, which generally last 10 to 15 years (Hartmann & Linn, 2008).

Our findings show that the annual per-household expenditure for basic program activities under *Jeevika* declined from approximately $34 per member when the program served<60,000 households, to almost $3 per member at its scaled-up level when the program reached more than 11 million households. Additionally, the results indicate larger economies of scale for the formation of higher-level federations, which is central to facilitating the impact of the *Jeevika* program (Kochar et al., 2020). We find that a 1 percent increase in the number of CLFs formed is associated with an increase in program expenditures of only 0.49 percent.

We also find that the scaled-up version of *Jeevika* may be as cost-effective in achieving benefits from reduced dependence on high-cost loans as its pilot version because of the magnitude of the economies of scale. We examined the question on how the cost-effectiveness of *Jeevika* could change with scale by comparing a cost-effectiveness ratio (CER) for benefits from reduced dependence on high-cost loans after an initial pilot of *Jeevika* with an estimate of the CER after the scale up of the program. While the scaled-up program may be equally cost-effective as the pilot in achieving benefits from reduced dependence on high-cost loans, the scaled-up program is less cost-effective than the pilot in achieving positive effects on women’s empowerment and asset ownership. As in the previous impact evaluations, this paper treats reduced dependency on formal loans as the primary outcome, but we also examined costs of producing potential downstream effects on women’s empowerment and ownership of consumption and productive assets. Unlike during the pilot, the evaluation of the scaled-up *Jeevika* program did not find statistically significant average effects on women’s asset ownership and intra-household decision-making (Datta, 2015, Hoffman et al., 2018). Yet, the evaluation of the scaled-up program showed evidence for heterogeneous effects, with landless households benefiting more from lower costs of borrowing, which led to increased asset ownership for this sub-group. Specifically for landless households, we found that the scaled-up program costed $160 for a one standard deviation increase in the consumption asset index, and $722 for a one standard deviation increase in the productive asset index.^1^

## 2 Conceptual framework

Considering a classical production function framework, *Jeevika* operates with economies of scale when average costs reduce with the number of women mobilized, and diseconomies of scale when average costs increase with the number of women mobilized.^2^ While standard microeconomic theory suggests lower average costs after an increase in scale, the magnitude of economies of scale for a program like *Jeevika* depends on the complexities of program delivery tied to the political economy of the implementation of government programs, including but not limited to government bureaucracy (Artuc et al., 2020), partnerships with NGOs and other government institutions, as well as the group-based nature of the program (Dìaz-Martin et al., 2020). The NRLM emphasizes partnerships with local NGOs and with Panchayati Raj Institutions (PRIs). As the nodal point at the district level, PRIs help in planning, coordinating, monitoring and regulating the implementation of national programs. Partnering with PRIs is therefore critical for social mobilization and formation of SHGs and federations, and especially for “convergence” with other government programs. As *Jeevika* scaled up, the program layered additional services like health and livelihoods trainings, which are often delivered through local NGOs and other Civil Society Organizations.

Political economy considerations imply that complex multi-faceted programs, such as *Jeevika*, require effective interagency coordination to achieve complementarities with other government programs and departments (Slater et al., 2016). A high number of policy actors may result in conflicting incentives (Lipsky, 2010, Pressman and Wildavsky, 1984), and implementation over an extended period may result in changing program priorities over time (Bruns et al., 2019). Complex multi-faceted programs also require more contextual information for effective implementation (Ricks & Doner, 2021), which could result in increased costs of targeting and adapting the programming to newly added geographies, especially with increased diversity at the district level. Additionally, scaling up to new districts is usually accompanied by changes in institutional capacity, which may lead to differences in implementation and effectiveness (Paul et al., 2021).

Pre-existing social and human capital may create additional barriers or facilitators for the functioning and scaling of SHGs. In the context of NRLM, scaling up is often accompanied with changes in implementation processes, including changes in the experience and education of community mobilizers. While early-stage SHGs in states where NRLM was newly implemented were usually formed by resource personnel brought in from states where SHGs were implemented sooner, such as Andhra Pradesh, the later formed SHGs were often developed by internal teams from the region. These new teams inevitably had less experience than the teams from more experienced states despite receiving additional training. Evidence also shows that SHG performance, and impacts, are higher when SHG women and their household members have higher education levels (Kochar et al., 2020). Finally, institutional theorists have posited that public programs have incentives to overspend, because expenditures are a direct visible outcome that stakeholders may perceive as the program’s success in the absence of impact data (Paul et al., 2021). Nonetheless, almost all government programs continue to have incentives to maximize concrete project outcomes within a budget constraint because of limited resources.

Looking at informal micro-level institutional factors, both costs and impacts depend on existing community norms and collective household preferences. Understanding local context is important to understand the external validity of the results (Deaton & Cartwright, 2018). Garikipati (2008) argues that a non-patriarchal hold on productive assets is a necessary condition for positive impacts of SHGs on women’s empowerment in India. An evaluation of the National Rural Livelihoods Project in seven states including Bihar further found that men’s education affects loan amounts drawn by SHGs, and their husband’s occupation explains women’s decision-making and labor force participation outside the household (Kochar et al., 2020). SHG programs also may have higher benefits for *a priori* more marginalized populations – for example, ST households in Andhra Pradesh (Prennushi & Gupta, 2014), and landless households (predominantly SC) in Bihar (Hoffman et al., 2018).

A recent study on the costs of economic inclusion programs found comparable average program costs of *Jeevika* in Bihar, and the NRLM which is implemented countrywide (Paul et al., 2021). Yet, we expect significant heterogeneities within states because of varying social norms and other contextual characteristics that may lead to different targeting and program adaptation costs. Participation in SHGs, as well as some of the intended outcomes, such as women’s mobility, often require the transgression of gender and social norms (De Hoop et al., 2014). Conducting outreach to maximize program participation and ensure program effectiveness may therefore require additional time and costs as shown in qualitative studies focusing on the implementation of *Jeevika*. In the initial stages of program mobilization, coordinators spent time collecting information on underlying community practices including caste dynamics, land use patterns, informal moneylending, and patriarchal norms, and changed their approaches for mobilization when they realized conflicts with existing norms and practices (Hoffman et al., 2018). For example, coordinators spent extensive time on negotiations and getting buy-in from male members and other members of the household and village, understanding the culture of caste-based handouts to differentiate *Jeevika*’s rollout form existing practices, and addressing community-specific concerns (Hoffman et al., 2018). These effective engagement processes would likely increase program costs when expanding to new diverse areas.

Another key institutional feature that may affect the magnitude of economies of scale of *Jeevika* is the group-based nature of the program. As indicated by Dìaz-Martin et al. (2020), SHGs and other women’s groups may achieve economies of scale because it is less costly to deliver training to the same number of women in a group setting than through individual home visits. In addition, collective action may generate larger impacts when groups enable members to gain access to new markets and services or members can achieve larger collective bargaining power (Anderson et al., 2019).

In this study, we start from the premise that *Jeevika* aims to maximize women’s social and economic empowerment at the lowest cost; or the program aims to minimize operational costs for each level of outcome. The extent to which the program achieves this objective depends, among other factors, on program scale. On the one hand, a higher number of women mobilized implies higher program exposure and a higher number of potentially empowered women. On the other hand, increasing the number of women mobilized may reduce the probability of achieving the outcome if program scale is negatively associated with program impact. An ethnography study indeed found substantial differences in the quality of implementation of *Jeevika* before 2012 when the program operated at a smaller scale, and after 2012 when the program scaled up across the state (Majumdar et al., 2017).

While economies of scale suggest that costs may decrease when programs expand beyond their initial outreach, the institutional factors listed above indicate that the scope of cost efficiencies depends heavily on institutional mechanisms. In subsequent sections, we study how average costs of the program changed with scale and shed some light on the implications for program cost-effectiveness.

## 3 Program background

Located in the Northern belt of India, Bihar is a primarily rural state, with 89% of its population residing in rural areas (Government of India, 2011), and has historically performed lower than other states on most social and economic development indicators. In 2012, the second largest share of the country’s poor lived in Bihar, which had a poverty rate of 34%, significantly higher than the national average of 22% (World Bank, 2018b). The state grapples with gender disparities in multiple domains. At just 9% and 15% respectively, the state has the lowest female labor force participation rate and the second lowest secondary education attainment rate among women in the country (World Bank, 2018b). Data from the National Family Health Survey-4 from 2016 further indicate that 33% of women in Bihar reported having “money that they can decide how to use” and 26% indicated having bank or savings accounts that they themselves use, compared to a national average of 42% and 53% respectively (International Institute for Population Sciences (IIPS) & ICF, 2017).

In 2005–06, with support from the World Bank, the Government of Bihar started planning for the launch of the Bihar Rural Livelihoods Project (BRLP). The structure and goals of the BRLP were defined along similar poverty alleviation projects as in other states, with an initial goal of improving livelihoods of the rural poor by developing institutional capacity through women’s SHGs and linking them to formal financial institutions and other agencies to negotiate better services. In 2007, the Government of Bihar established the *Jeevika* in six priority districts of the state with the aim of mobilizing poor households into SHGs under the BRLP. As a result of the NRLM launch and with additional funding from the World Bank in 2011–12, *Jeevika* expanded its services and the scale of SHGs to cover a greater number of districts, and by 2021, had mobilized over eleven million households.

The federated structure of SHGs under *Jeevika* facilitates collective action, adoption of livelihoods enhancement and income generating activities, and development of linkages with market institutions (World Bank, 2017). A typical *Jeevika* SHG consists of 10 to 15 women who come together as a platform for accessing program services that include access to low-cost credit and formal banking, as well as basic literacy and livelihoods training (Hoffman et al., 2018). In the second tier, SHGs are federated into VOs, which are further federated into CLFs at the third level. The time between SHG formation and formation of VOs or CLFs may vary considerably, and depends on regularity of meetings, savings, and repayment, and the formation of a microcredit plan. These baseline performance indicators also affect the time between SHG formation and bank linkage of the SHG. Additionally, the delivery of these program activities depends upon the local capacity of the VO and CLF formation teams. For example, studies found that across seven states that implemented the NRLM, there was a shortage in staff for VO formation, resulting in delays before SHGs were linked to federations (Kochar et al., 2020).

Program expenditures under *Jeevika* are reported for four broad components: Community Institution Development (CID), Special Technical Assistance Fund (STAF), Project Management (PM), and the Community Investment Fund (CIF). The CID fund provides all expenses for institution building, including the costs of mobilizing groups, developing direct linkages to the formal finance services providers, formation of federations, and capacity building of staff at the district, block, and cluster level. The STAF provides technical assistance to the formal financial sector and other partners to support microfinance initiatives and pro-poor banking and promote public–private partnerships to improve the quality and quantity of public services delivered to communities. Overall coordination, project implementation, financial management and monitoring and evaluation at the state and district levels are supported through resources under the PM component. Finally, the CIF component provides direct grants to the community through the Revolving Funds (RFs), as well as through Vulnerability Reduction Funds (VRFs). The RF aims to meet the credit need of group members and is considered “catalytic capital” for leveraging repeat bank finance, thus promoting income generating activities, for example through investments in livelihood skills development. The VRF is usually provided to VOs to address food security risks, health risks, and natural disasters through the Food Security Fund or Health Security Fund. *Jeevika* disburses CIF to SHGs and federations as a grant on a demand-driven basis through a participatory micro-planning process.

## 4 Data

### 4.1 Cost data

We obtained data on program implementation, components, outreach, and expenditures from *Jeevika* annual reports and audited financial statements from 2007–08 to 2018–19. These data provide information on annual program outreach and scale, including the number of households mobilized into SHGs, number of SHGs promoted, number of VOs and CLFs formed, and the number of SHGs linked to a bank account. We collected data on program expenditures from annual audit statements of *Jeevika* and World Bank project documents. It is important to note that our analyses are only based on reported program expenditures. Ideally, a costing analysis should include costs of resources that were utilized but not directly paid for – for example, the time spent by volunteer staff, or use of buildings and resources for BRLP-specific activities that may have been already in use and paid for by another government entity. All costs presented in this study should therefore be considered as an underestimate of true program costs and should be regarded as program accounting costs only. For this reason, we should also exercise some caution in the interpretation of the cost-effectiveness estimates. We use the terms “costs” and “expenditures” interchangeably in the rest of this paper.

### 4.2 Impact data

We use findings from impact evaluations of the *Jeevika* program to complement our costing analysis and generate evidence on cost-effectiveness. We accessed and analyzed data from two impact evaluations of the *Jeevika* program – the first focusing on the pilot phase of the project pre-2012 (Datta, 2015) and the latter on the second scaled-up phase after 2012 (Hoffman et al., 2018, Hoffmann et al., 2021). Where needed, we complemented the estimates from the published literature with data posted on Harvard Dataverse (Datta and Rao, 2018a, Datta and Rao, 2018b, Datta and Rao, 2018b). The phase 1 survey dataset included 3,997 observations, 50% of whom belonged to treatment areas where *Jeevika* entered in 2008. The phase 2 dataset included 8,988 observations, 50% of whom belonged to treatment areas where *Jeevika* entered in 2012.

## 5 Methods

We examine the costs of increasing both the scope as well as the scale of services related to expanding *Jeevika*’s outreach of the SHG program. To analyze program scale, we consider costs from expanding the geographic supply of the program by adding more participants over time across the state. To analyze program scope, we study component-specific costs of the program, differentiating between expenditures related to key program activities (community mobilization and financial inclusion), and expenditures related to overall operations and management. We also study the changes in total program costs in relationship to core program activities, including the federation of SHGs, such as the formation of SHGs, VOs, CLFs, and bank linkages.

We estimate average program costs as costs per SHG woman targeted under the *Jeevika* program, treating each *Jeevika* member added as an additional program output. While not all women received the same level of services with the same intensity, we estimate an average cost equivalent to an “Intent to Treat” cost – that is, expenditure per woman SHG member with access to potential program services. From *Jeevika*’s expenditure components discussed earlier, we consider three expenditure components – Community Institution Development, Special Technical Assistance Fund, and Project Management. We do not include Community Investment Funds when studying the relationship of costs with scale because it is disbursed to SHGs or their federations as a one-time grant or capital based on need and is usually a fixed amount (of INR 66,000, on average, [Kochar et al., 2020]).^3^

*Jeevika* aims to form SHGs that will eventually become self-reliant and self-managed by gradually adopting income generating activities and profits for the federations. In 2017, the World Bank conducted an implementation assessment of the program, and through a random stratified sample, found that 89% of older SHGs (formed prior to 2012) had become self-reliant by 2017 (World Bank, 2017). Although we are unable to obtain precise estimates on SHGs that ceased to receive *Jeevika*’s support on different activities in every year, we estimate two cost models following different assumptions around the definition of scale or size of the target groups for these activities – (1) Model 1 – Cumulative Participation, under which we assume that every SHG member under *Jeevika* is eligible for all services, irrespective of when they joined the program; and (2) Model 2 – Fixed-Period Participation, under which we assume that *Jeevika* supports all SHGs and related-activities for a period of three years, after which SHGs become self-sustainable. For Model 2, we divide all expenditure components in each year among women who have been a member of *Jeevika* for no longer than three years. In our conversations with the program implementation team, we were informed that *Jeevika* continues to support participants throughout their membership, and therefore the first assumption is more plausible. The nature of support is likely to vary over time, however, with older groups receiving more support with economic livelihoods and newer groups receiving support with bank and credit linkages.

We extracted all program expenditure data in local currency (INR) and converted to 2018 USD by first converting annual expenses to 2018 prices by adjusting for INR inflation using the Consumer Price Index (CPI) method, and then converting to 2018 USD by adjusting for 2018 market exchange rate. We then used the production function to test the possibility of increasing, decreasing or constant returns to scale, for the same program over time. We estimated the time-varying growth in logged costs as a function of logged scale as shown in Eq. (1):

$$L\mathrm{og}{\left(TC\right)}_{t}=\alpha_{0}+\alpha_{1}Log\left(n_{t}\right)+{∊}_{t}$$

In Eq. (1), $\mathrm{Log}{\left(TC\right)}_{t}$ is the logged program costs in year t; and $n_{t}$ is the program scale in year t. $\alpha_{1}$ is the parameter of interest and shows the percentage change in costs for a given percentage change in program scale. If 0< $\alpha_{1}<1$, the model suggests that as the program scales up, the increase in total costs is lower than the increase in scale, and we find evidence for economies of scale. The log transformation of all indicators lends an easy interpretation for the question at hand (Bikker et al., 2012) and helps address concerns about heteroskedasticity and non-stationarity.^4^ It is important to note that our regressions cover twelve years of data, which is indisputably a small sample for a formal regression analysis. Yet, costing and cost-effectiveness analyses rarely include data observed at more than one time-point because of data limitations. The time covers the full period of program implementation, and the period is typical for usual scaling-up processes (Hartmann & Linn, 2008). The goal of our analysis is to provide insight into how costs vary as program scale varies, which previous studies have carried out with much smaller sample sizes (see for example Tulloch, 2019, who uses seven observations).

Next, we investigate how total program expenditures change with respect to specific economic activities. We use four different indicators of economic activities under *Jeevika*, focusing on the primary program outcomes – (1) annual SHG formation; (2) annual VO formation; (3) annual CLF formation; and (4) annual number of SHGs linked to the bank. The first three activities directly address the key output of *Jeevika* – formation of women-led community institutions at different levels. The fourth activity denotes the financial inclusion goal of the program. Our data do not allow for disaggregating costs beyond the three categories of CID, PM, and STAF, implying that we cannot reliably estimate the cost of each specific activity. Yet, following the same specification as in Eq. (1), we can estimate how the total annual expenditure of program changes with respect to each of the four indicators. To do this, we replace $n_{t}$ in Eq. (1) with the scale of specific program activities, as shown in Eq. (2) below:

$$\mathrm{Log}{\left(TC\right)}_{t}=\alpha_{0}+\alpha_{1}Log\left(X_{t}\right)+{∊}_{t}$$

We estimate four different specifications of Eq. (2) for each economic activity as input for $X_{t}$ (as opposed to one regression with the four activities because of high correlations across the activities). We compare $\alpha_{1}$ across the four activities to understand differences in economies of scale across economic activities.

Finally, we examine whether the pace of achieving program outputs and likelihood of impacts changes with scale using two different approaches. We start by leveraging the data on program activities to analyze two key channels through which the program aims to improve women’s economic outcomes – financial inclusion and federation formation. Financial inclusion, including access to and use of formal banking services, is the first program focus after group mobilization. If scaling up adversely impacts implementation quality, many SHGs may become defunct, women may stop attending group meetings, and the pace of linking SHGs to formal bank accounts may slow down. Further, formation of higher-level federations depends on additional program capacity on the implementation side, and a certain ‘grade’ of performance on the SHG side (Kochar et al., 2020). Federations provide a range of benefits, including trainings for livelihoods services, serving as an interface between SHGs and government programs, and providing women with a collective voice for access to entitlements. Therefore, we next examine the relationship of scale with – (1) the pace of SHG linkages with bank accounts (using logged number of SHGs linked to bank as the outcome); (2) the pace of VO formation (using logged number of VOs as the outcome); and (3) the pace of CLF formation (using logged number of CLFs as the outcome). While these program outputs only serve as *channels* for ultimate program impacts, the key advantage of analyzing these data is that, unlike impact evaluation data, data on performance indicators are available annually and therefore provide more data points to create a time-series dataset.

Second, we use two existing impact evaluations to estimate program cost-effectiveness during the pilot and scaled-up phases (Datta, 2015, Hoffman et al., 2018, Hoffmann et al., 2021). We primarily focus on the most direct source of program impact by examining household-level cost savings from borrowing at lower interest rates. Studies have shown that *Jeevika* resulted in a statistically significant and sizeable reduction in interest rates on loans taken by households, primarily due to reduced dependence on informal moneylenders (Hoffmann et al., 2021).^5^ Although we consider financial inclusion as the primary outcome, the program could create downstream effects on economic outcomes, such as consumption and productive asset ownership, as well as women’s empowerment. Therefore, the cost-effectiveness for financial inclusion may present an incomplete picture of the overall cost-effectiveness of the program. To address this concern, we also discuss the program effects and cost-effectiveness for intra-household decision-making outcomes and asset ownership, including heterogeneous effects on asset ownership for landless households. However, the data do not allow for a comparative analysis for these outcomes at pilot versus scaled-up phases, because of the differences in their measurement across the two impact evaluations, and because the pilot phase does not report heterogeneous effects for landless households. Cost-effectiveness ratios were estimated by dividing the costs during program participation years by program impacts.

## 6 Results

### 6.1 Program scale

**Fig. 1:**
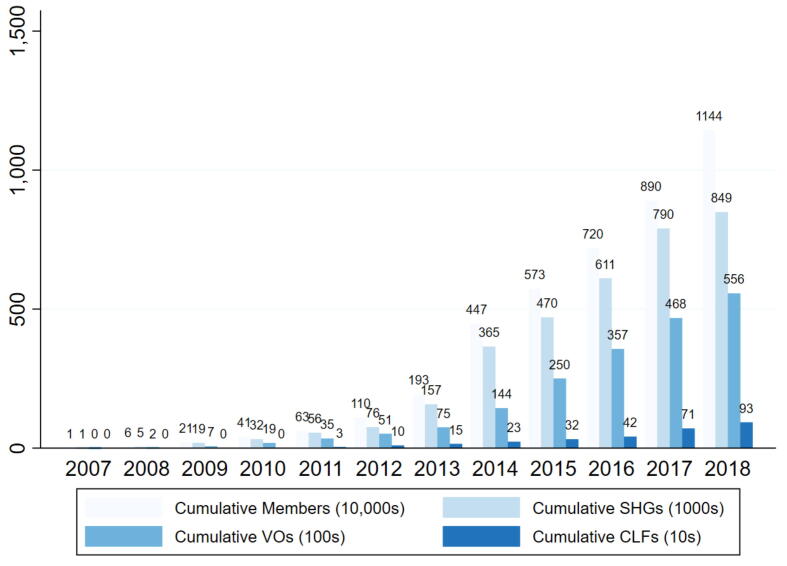
Jeevika Program Scale-Up. Note: The x-axis depicts the start of financial year, and ranges from 2007 to 08 to 2018–19.

We begin the analysis with an overview of the scale of *Jeevika*’s operations between 2007–08 to 2018–19. Figure 1 shows the number of SHGs, VOs, and CLFs formed by the end of each financial year, indicating a steep increase in mobilization after 2012–13. In 2011–12, *Jeevika* took over the expansion of SHGs under the NRLM and received additional financing from the World Bank for the second phase of the BRLP project. Reports suggest that the initial two years of expansion were relatively challenging because of delays in recruiting adequate staff (World Bank, 2017). However, as shown in Figure 1, the pace of mobilization increased steadily post expansion, especially after 2012–13, and by 2018–19 over 11.4 million women had been mobilized into almost half a million SHGs.

### 6.2 Program costs

**Fig. 2:**
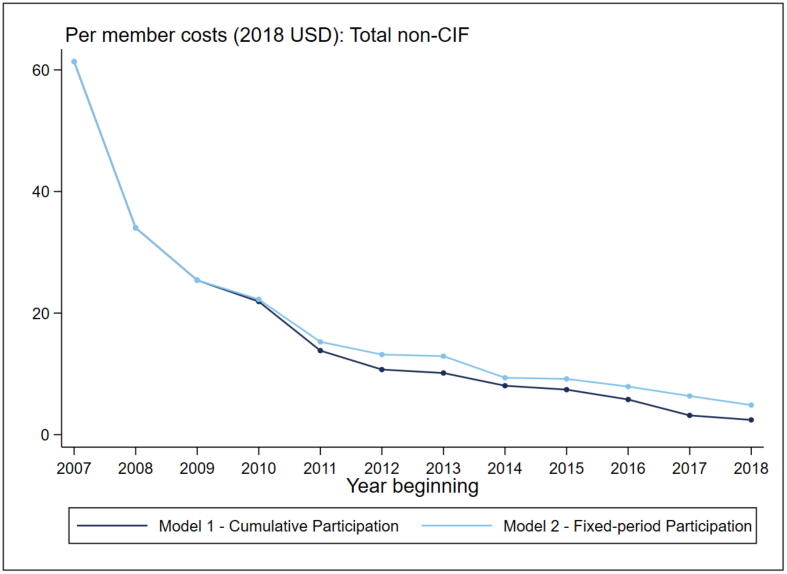
Jeevika Annual non-CIF Expenditure Over Time. Note: The x-axis depicts the start of financial year, and ranges from 2007–08 to 2018–19. Y-axis shows per member combined annual expenditure on Community Institutional Development, Special Technical Assistance Fund, and Project Management. Model 1 assumes that expenditures are divided across all members irrespective of when they joined the program. Model 2 assumes that all members are supported for three years after joining the program.

Next, we analyze annual expenditure related to basic SHG programming and program operations. Overall, we find a steep decline in program expenditures per member on basic group activities in initial years of the program, with the decline becoming slower after 2011–12. This change overlapped with the large increase in program scale during the second phase of the project. These expenditures include amount spent on activities like mobilization, group meetings and financial inclusion (CID), project management (PM), and technical assistance (STAF). Figure 2 shows the change in per member expenditure on these activities under two assumptions – (1) assuming that cumulative number of program beneficiaries are eligible for support in a given year (Model 1); and (2) assuming that all beneficiaries are eligible for program support for up to three years from the year of initial participation (Model 2). By 2018–19, with a membership of 11.4 million beneficiaries, the annual per member expenditure ranged between $2.5 in Model 1 and $4.9 in Model 2.

Table 1 presents the results more formally using regression estimates of the cost function. Our results suggest that a 1% increase in *Jeevika* membership was associated with a <1% increase in program costs, suggesting strong economies of scale. Specifically, a 1% increase in scale was associated with a 0.6% increase in expenditures in Model 1, and a 0.7% increase in expenditures in Model 2. Additionally, the marginal change in costs decreases with increases in outreach. Specifically, the estimates suggest that annual per capita expenditure was approximately $29.6 in Model 1 and $29.1 in Model 2 when the program served 100,000 women; around $11.8 in Model 1 and $13.3 in Model 2 when the program served one million women; and fell as low as $4.7 in Model 1 and $6.1 in Model 2 when the program served ten million women. These predicted costs suggest that the decline in costs per capita slows down with a large increase in scale. This is likely because as scale increases by a large number, existing inputs may reach their threshold of productivity and the program may require a large amount of new inputs, which drives up the operational costs.

**Table 1:** Jeevika Total Annual non-CIF Expenditure.

	(1)	(2)
VARIABLES	Model 1 All non-CIF costs (logged)	Model 2 All non-CIF costs (logged)
Logged total outreach	0.601***	0.660***
	(0.046)	(0.028)
Constant	7.938***	7.270***
	(0.606)	(0.359)

Observations	12	12
R-squared	0.959	0.983
F test:	172.5	562.2

*Model predictions*		
Average cost with 100,000 members	29.60	29.13
Average cost with 1 million members	11.80	13.31
Average cost with 10 million members	4.71	6.08

Robust standard errors in parentheses. *** p < 0.01, ** p < 0.05, * p < 0.1. Model 1 assumes that expenditures are divided across all members irrespective of when they joined the program. Model 2 assumes that all members are supported for three years after joining the program. Breusch-Godfrey test statistics for serial autocorrelation: (1) Cumulative participation: F-statistic = 3.87; p-value = 0.08; (2) Fixed-period participation: F-statistic = 3.37; p-value = 0.10. Both models fail to reject the null hypothesis of no serial correlation.

**Fig. 3:**
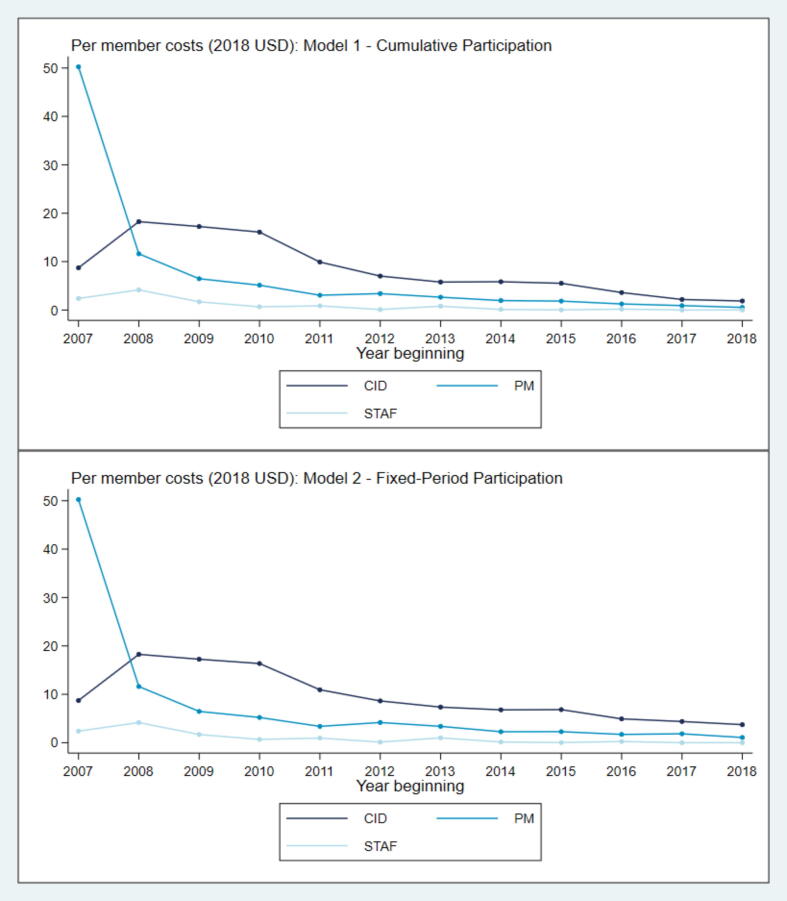
Jeevika non-CIF Expenditure Components Over Time. Note: The x-axis depicts the start of financial year, and ranges from 2007–08 to 2018–19. Y-axis shows per member annual expenditure by expenditure component. CID: Community Institutional Development; STAF: Special Technical Assistance Fund: PM: Project Management. Model 1 assumes that expenditures are divided across all members irrespective of when they joined the program. Model 2 assumes that all members are supported for three years after joining the program.

Next, we break down the overall non-CIF expenditure into its subcomponents – specifically, CID, STAF, and PM related expenditure. As shown in Fig. 3, CID expenditure per member was highest in the initial years (at $17.35 per capita), kept declining after 2009, and the decline tapered off after 2012. At the maximum program outreach of 11.4 million members, CID expenditure was between $1.8 (under Model 1) and $3.7 (under Model 2) per capita. The relative share of CID expenditure became closer to PM and STAF related expenditure in the later years of program. STAF costs changed from $3.95 per capita when the project had an outreach of 59,000 members to 17 cents per member when the project had the highest outreach of 11.4 million members. Average expenditure related to PM activities also declined rapidly over time. In the first year of the project, PM expenditure was estimated at $47 per capita, which declined to $0.01 under both Models 1 and 2 by the latest year (2018–19).

The CID component includes expenditures on project start-up, community mobilization, formation of higher-level federations, capacity management, and implementation of inclusive strategies to ensure that the project activities prioritize the most economically and socially deprived communities. Initially, as the program scaled and mobilized more households, a higher proportion of resources was devoted to CID (between years 2008 and 2011). STAF related costs were used to cover expenses related to program innovation, partnerships with government agencies and non-governmental organizations for financial inclusion and technical support on production and livelihoods activities. The project reported having spent lower amounts on the STAF component than initially estimated because of leveraging already established innovation linkages (World Bank, 2017). The inverse U-shaped trend in the relationship between per-member annual CID as well as STAF expenditure and scale suggests increasing expenditures after an increase in the number of households covered when the program is at a relatively small scale (under 200,000 households), and a decrease in costs as the program scales up using its established infrastructure and capacity-building network. Finally, the initial peak in project management expenses may indicate high investment in project implementation support activities in the initial years, including monitoring, evaluation and learning progress and setting up of the project Management Information System.

More formally, we find evidence for economies of scale across all three expenditure components, as shown in regression estimates in Table 2. An increase in outreach by 1% was associated with the largest cost savings in annual STAF expenditure (an increase of 0.4%; p < 0.05), followed by PM-related expenditure (an increase of 0.5%; p < 0.01), while economies of scale were slightly lower for CID expenditure (an increase of 0.7% to 0.8%; p < 0.01). These estimates indicate that average costs at a scale of 100,000 women were $12.9 to $13.6 in annual CID expenses, $2.3 in annual STAF expenses, and $10.9 to $11.0 in annual PM expenses. The same models predict that when the program operated at a scale of 10 million women, the annual per capita expenses reduced to $4.4 to $5.9 per program participant for CID, $0.1 per program participant for STAF, and $1.0 to $1.2 per program participant for PM related components.

**Table 2:** Jeevika Annual non-CIF Expenditure Components.

	(1)	(2)	(3)	(4)	(5)	(6)
VARIABLES	Model 1 CID costs (logged)	Model 2 CID costs (logged)	Model 1 STAF costs (logged)	Model 2 STAF costs (logged)	Model 1 PM costs (logged)	Model 2 PM costs (logged)
Logged total outreach	0.753***	0.831***	0.405**	0.426**	0.478***	0.522***
	(0.106)	(0.091)	(0.169)	(0.174)	(0.034)	(0.036)
Constant	5.307***	4.426***	7.142**	6.921**	8.364***	7.876***
	(1.526)	(1.308)	(2.236)	(2.277)	(0.431)	(0.495)

Observations	12	12	10	10	12	12
R-squared	0.920	0.951	0.456	0.460	0.950	0.962
F test:	50.93	82.94	5.743	5.957	198.9	206.4

*Model predictions*						
Average cost with 100,000 members	13.57	12.94	2.30	2.33	10.95	11.02
Average cost with 1 million members	7.69	8.76	0.59	0.62	3.29	3.67
Average cost with 10 million members	4.36	5.93	0.15	0.17	0.99	1.22

Robust standard errors in parentheses. *** p < 0.01, ** p < 0.05, * p < 0.1. Model 1 assumes that expenditures are divided across all members irrespective of when they joined the program. Model 2 assumes that all members are supported for three years after joining the program. Breusch-Godfrey test statistics for serial autocorrelation: CID costs (1) Cumulative participation: F-statistic = 1.68; p-value = 0.23; (2) Fixed-period participation: F-statistic = 1.38; p-value = 0.27. STAF costs (1) Cumulative participation: F-statistic = 1.41; p-value = 0.27; (2) Fixed-period participation: F-statistic = 1.49; p-value = 0.26. PM costs (1) Cumulative participation: F-statistic = 2.01; p-value = 0.19; (2) Fixed-period participation: F-statistic = 0.37; p-value = 0.56. All models fail to reject the null hypothesis of no serial correlation. For the final two years, *Jeevika* did not report any STAF expenditure.

### 6.3 Scale and costs

We next analyze how annual program expenditures change in response to changes in annual (1) SHG formation, (2) VO formation, (3) CLF formation, and (4) the number of SHGs linked to the bank. Table 3 shows that a 1% increase in each of the four activities considered was associated with a <1% increase in annual program expenditure – indicating economies of scale across the board. The number of CLFs formed was associated with the largest gains, and SHG formation was associated with the least gains in scale economies. Specifically, annual program expenditures increased by 0.49% with a 1% increase in the number of annual CLFs formed, and by 0.77% with a 1% increase in the number of new SHGs formed. This result may reflect the fact that SHG formation is the first program activity as part of community mobilization, while CLF formation occurs once groups have been functioning for a period of time, have formed VOs, and have established the required capacity and infrastructure. Interestingly, we also find a high degree of economies of scope for the number of SHGs that are linked to formal credit.

**Table 3:** Logged Total Annual Expenditure as a Function of Different Economic Activities.

	(1)	(2)	(3)	(4)
	Total cost (2018 USD) Logged	Total cost (2018 USD) Logged	Total cost (2018 USD) Logged	Total cost (2018 USD) Logged
Logged SHGs formed	0.769***			
	(0.049)			
Logged SHGs linked to credit		0.542***		
		(0.048)		
Logged VOs formed			0.709***	
			(0.033)	
Logged CLFs formed				0.495***
				(0.138)
Constant	8.311***	11.004***	10.935***	14.743***
	(0.492)	(0.445)	(0.239)	(0.616)

Observations	12	12	12	12
R-squared	0.947	0.940	0.977	0.667
F test:	242.6	129.5	457.8	12.83

Robust standard errors in parentheses. *** p < 0.01, ** p < 0.05, * p < 0.1. The table includes estimates from four different regressions, each with the same outcome (total annual cost, logged) and different independent variables for each of the four economic activities.

### 6.4 Scale and outputs

We next analyze how three key performance indicators changed with respect to scale. Our estimates suggest that the pace of project outputs increased significantly with program scale (Table 4). Specifically, a 1% increase in program scale was associated with a 1.3% increase in the number of SHGs with a bank account, a 1.1% increase in the number of VOs, and a 1.1% increase in the number of CLFs. While the estimates suggest positive returns to scale, the gains are empirically small, suggesting no strong gains or losses from scaling up in terms of these program performance indicators. These indicators only reflect the pace of project activities, and not necessarily the *quality* of project activities, however. As discussed below, these performance indicators likely present an incomplete picture of the change in full benefits, for example, in case linkages to federations are equally likely for all SHGs but new federations produce smaller impacts because of their weaker capacity.

**Table 4:** Program Scale & Key Outputs per SHG.

	(1)	(2)	(3)
VARIABLES	Logged credit linked SHGs	Logged VOs	Logged CLFs
Logged total outreach	1.257***	1.060***	1.123***
	(0.040)	(0.019)	(0.208)
Constant	−7.020***	−6.324***	−11.878***
	(0.566)	(0.280)	(3.138)
Observations	12	12	12
R-squared	0.989	0.994	0.815
F test:	1009	2974	29.19

Robust standard errors in parentheses. *** p < 0.01, ** p < 0.05, * p < 0.1. The table includes estimates from three different regressions.

## 7 Implications for cost-effectiveness

Our findings suggest that large scale implementation of women’s SHG programs has considerable potential to benefit from reduced costs due to economies of scale. These findings produce an important side to an often-told incomplete story where successful pilot interventions fail to produce similar impacts when scaled up (Vivalt, 2020; Cull & McKenzie, 2020). Considering the relationship between program impact and scale in isolation from the relationship between program costs and scale could thus mask a crucial element of translating results to policy recommendations and action and may even result in misleading policy implications.

Findings from two impact evaluations (Datta, 2015, Hoffmann et al., 2021) – the first focusing on the first pilot phase of the project (pre-2012) and the latter on the second phase during scale-up (2012–2014) – indicated that while *Jeevika* was able to generate strong positive effects during the pilot phase, especially on social empowerment, the second phase (post-2012) failed to see most of these individual-level and household-level effects. These changes in impacts likely result from the scale and complexity of the *Jeevika* program. The program includes multiple components, covers a wide geographical area, and as of 2021, covers over eleven million households. The implementation of the program also varies across contexts due to variation in local capacity, population needs, and other contextual factors. Further, as the program scaled up, several components of the program were implemented in collaboration with different organizations.

An ethnographic study attributed the failure to produce similar effects in the second phase to the pressure to scale up quickly (Majumdar et al., 2017). The study had three primary findings – (1) During the second phase, program participants were mobilized quickly with a fixed script, unlike the first phase which involved creative improvisation that involved multiple community stakeholders; (2) Community facilitators lacked experience and drive in the second phase having spent little to no time on institutional learning; and (3) Limited resources in the second phase meant little room for slow learning, and lower investments in the form of capitalization funds.

Combining our cost estimates with existing impact evaluation estimates, we find that despite the considerably lower impacts on high-cost loans of households after the scale-up than during the pilot phase, the differences fade away when we estimate cost-effectiveness ratios. For the cost-effectiveness analysis, we first focus on the costs of achieving benefits related to lower costs of borrowing, estimated as the ratio of costs to household change in high-cost debt. In Phase 1, the program led to a 46% decrease in total high-cost debt per household, while in phase 2, the decrease was approximately 15% (Majumdar et al., 2017, Datta, 2015, Hoffman et al., 2018).^6^ As shown in Table 5, we find an interesting pattern in the cost-effectiveness ratios (CERs) for high-cost debt. Before 2012, that is in phase 1 of the project, the average high-cost loan amount decreased by $89 because of the *Jeevika* program, more than twice the average impact of $33 in phase 2. However, it costed 91 cents to reduce each additional dollar of high-cost debt in phase 1 – only slightly different from 88 cents in phase 2. While program impact declined by almost 62% after scale-up, it was 3 cents less costly after scale-up to achieve an additional dollar of benefit in reducing high-cost debts than during the pilot.

**Table 5:** Cost Per Dollar Change in Outcomes.

	Phase 1	Phase 2
***Primary costs, outcomes, and cost-effectiveness***		
Average change in high-cost loan amount per household	89.08	32.83
Operating costs per capita	81.33	28.90
Cost per dollar of decrease in high-cost loan	0.91	0.88

***Empowerment impacts and cost-effectiveness in Phase 1***		
Cost per percentage-point increase in women attending Panchayat meetings	40.67	No significant impact on women’s empowerment index
Cost per percentage-point increase in women participating in health decisions	27.11	
Cost per percentage-point increase in women visiting health center	27.11	
Cost per percentage-point increase in women having an opinion on politics	20.33	
Cost per percentage-point increase in women providing input on children's education	81.33	

***Asset ownership impacts and cost-effectiveness for landless households in Phase 2***		
Costs per SD increase in consumption asset index	Not measured	160.57
Costs per SD increase in productive asset index		722.56

*Note*: All costs and high-cost loan amounts were converted to 2018 USD by first adjusting for Rupee inflation using the CPI method, and then converting to 2018 US Dollars using the Market Exchange Rate. Assumptions include a uniform average loan amount of Rs.10,000 in both phases for both treated and control groups, estimated from Hoffman et al. (2018) and Datta et al. (2015). SD = standard deviation.

**Fig. 4:**
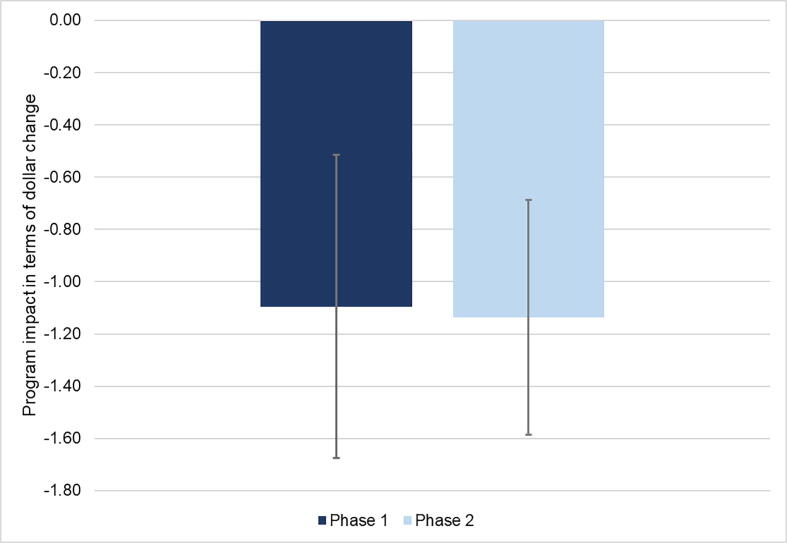
Change in High-Cost Loan Amount for Every Dollar Spent on the Program. Note: All estimates are in 2018 USD. Error bars represent 95% confidence intervals. Costs are based on Model 1 assumptions –expenditures are divided across all members irrespective of when they joined the program.

In line with Evans and Popova (2016), we also present confidence intervals for additional benefits per dollar spent on *Jeevika* to assess the uncertainty in cost-effectiveness estimates due to imprecision in the impact estimates. Figure 4 shows that every dollar spent on the program reduced the high-cost loan amount by $1.10 in Phase 1, and by $1.14 in Phase 2. The 95% confidence intervals show that the lower bound is only slightly higher in Phase 2 while the upper bound is slightly higher in Phase 1. Overall, we do not find any meaningful difference in the confidence intervals for program cost-effectiveness over the two phases.

Some caveats should be considered when interpreting these findings. First, the Phase 1 evaluation is less robust as it relied on propensity score matching on retrospective data, while the phase 2 evaluation used a randomized controlled trial. Therefore, impact estimates from Phase 1 evaluation may be an overestimate of the true program effect if motivated women were more likely to participate in the program. Second, the cost-effectiveness ratios shown here only apply to benefits caused by reduced costs of borrowing resulting from reduced interest rates. For this study, our primary aim is to demonstrate the consequences of ignoring cost data when interpreting findings of impact evaluations of scaled-up programs. Several other outcomes need to be considered in the estimation of a holistic benefit-to-cost ratio.^7^ The Phase 1 evaluation showed that the program led to significant improvements in women’s empowerment outcomes including mobility, decision-making, and propensities toward collective action; while the Phase 2 evaluation showed only weak and mostly conflicting effects on empowerment indicators.

However, the Phase 2 evaluation did show meaningful and statistically significant negative effects on interest rates charged by informal lenders, which resulted in a reduction in the difference in average borrowing rates faced by landholding and landless households by two-thirds (Hoffmann et al., 2021). The reduction in informal interest rates and the shift towards loans from formal sources both contributed to reducing the costs of borrowing, especially for landless households that had higher costs of borrowing at baseline. These impacts potentially led to other effects – the study showed statistically significant positive impacts on consumption asset and productive asset indices for landless households, despite very modest average treatment effects on consumption assets and null average treatment effects on productive assets for the full sample (Hoffman et al., 2018). Considering the wide reach of informal moneylenders among non-program participants, aggregate program benefits may increase even more following the program scale-up.

Current estimates of the cost-effectiveness of the scaled-up program for landless households indicate that it would cost $161 to increase the consumption asset index by one standard deviation, and $723 to increase the productive asset index by one standard deviation (Table 5). The reduced interest rates thus also seem to result in cost-effectiveness for other economic outcomes, at least for marginalized populations. However, the scaled-up program did not seem to achieve cost-effective improvements in consumption and women’s empowerment – neither for the full sample nor for landless households, since the effects were negligible and insignificant for these outcomes. At the same time, we find evidence that the pilot phase resulted in a larger cost-effectiveness of *Jeevika* in achieving positive effects on women’s empowerment outcomes (Table 5), indicating that prioritizing program elements that led to positive effects on asset ownership and women’s empowerment before the scaling may result in more positive cost-effectiveness for these outcomes even when costs are higher. Yet, given that average costs after the scale-up were half the costs compared to the pilot, the program could have retained the same levels of cost-effectiveness on empowerment and asset ownership if the scaled-up program had produced half the impacts as during the pilot.

## 8 Comparison with other programs

**Fig. 5:**
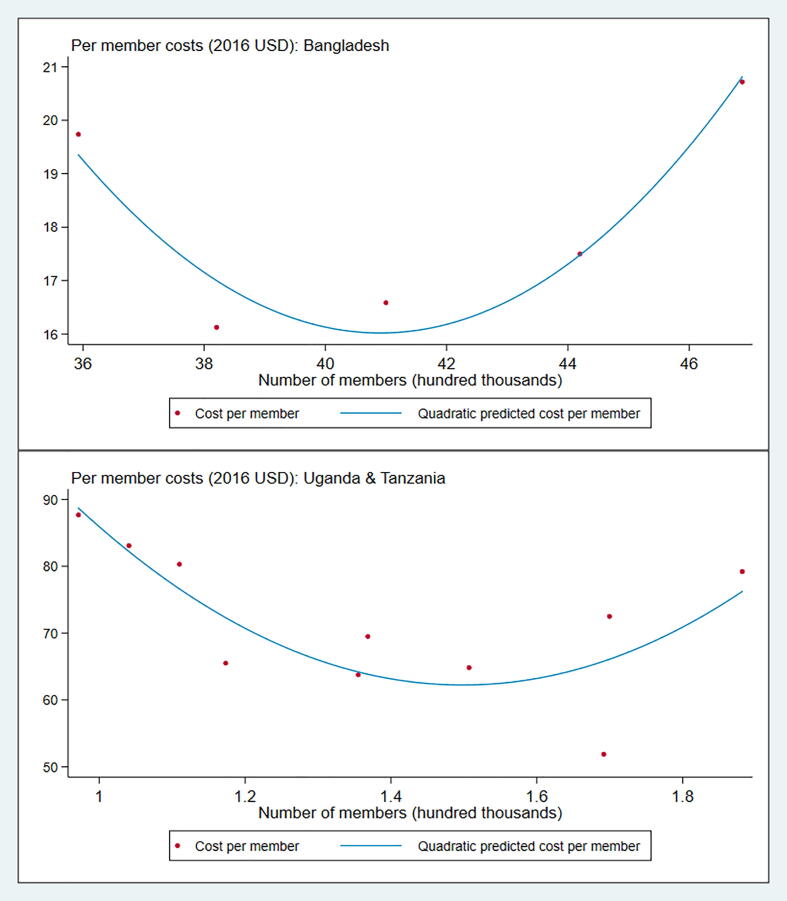
BRAC Microfinance Costs: 2013 to 2017. Note: The chart shows per member annual expenditure on y-axis, and number of members on x-axis. Bangladesh is shown separately from Uganda and Tanzania because of the wide disparity in scale. Scatter plots with polynomial fit lines are shown in both charts.

As a secondary analysis and to substantiate our findings on cost-efficiencies as a function of program scale, and to assess the external validity of the results, we used annual data on program expenditures and outreach of BRAC’s savings group-based microfinance program in Bangladesh, Uganda, and Tanzania. We used these data as a robustness check because of data availability but also because BRAC’s microfinance programs are amongst the largest women’s group programs with economic objectives in the world and operate across several countries. BRAC (originally Bangladesh Rehabilitation Assistance Committee) is among the world’s largest non-profit organizations with a scale of over 120 million people across eleven countries (BRAC, n.d.). BRAC’s groups-based microfinance program, which offers group-based loans delivered exclusively to women, started in Bangladesh in the 1970 s, and by 2017, had reached over eight countries. We collected data on operating costs per loan disbursement between 2013 and 2017 in Bangladesh (where the program had been operating for a long time and at a large scale), Uganda, and Tanzania. Figure 5 shows the operational costs per borrower with respect to total number of borrowers in the three countries. Between 2013 and 2017, average cost per borrower was $18.13 in Bangladesh, $70.67 in Uganda, and $72.97 in Tanzania. At the same time, BRAC microfinance had a much larger scale in Bangladesh, which is not surprising given that BRAC has operated in Bangladesh for almost four decades, while the operations in Tanzania and Uganda started much more recently in 2006.^8^

To analyze returns to scale, we examined how cost per borrower changed with the number of borrowers in each year (a proxy for microfinance outreach). Table 6 shows the formal regression estimates. The bivariate relationship between scale and costs (Column 1) indicates that an increase in member outreach of 100,000 persons is associated with a $1.3 decline in operational costs per borrower (p < 0.01). Column 2 adds the quadratic outreach term and shows that the decline in costs with respect to scale now increases to $3.1 for every 100,000 additional persons, but the decline slows down by $0.04 (although statistically insignificant). In column 3, we find that after controlling for country fixed effects, an additional 100,000 persons is associated with a decline in per member cost of $20, and that the decline slows down by $0.25 per 100,000 increase in outreach. The regressions show a qualitatively similar pattern as in the costing analyses of the *Jeevika* program, suggesting that the results may well be externally valid.

**Table 6:** BRAC Microfinance: 2013 to 2017.

	(1)	(2)	(3)
	Cost per borrower	Cost per borrower	Cost per borrower
Number of borrowers (in 100,000)	−1.328***	−3.147**	−20.345*
	(0.132)	(1.133)	(11.119)
Number of borrowers (in 100,000) squared		0.042	0.247*
		(0.026)	(0.135)
Country FEs: Tanzania			−335.777
			(216.228)
Country FEs: Uganda			−331.387
			(212.589)
Constant	73.400***	76.144***	432.986*
	(3.152)	(3.424)	(229.380)

Observations	15	15	15
R-squared	0.887	0.907	0.925
Includes quadratic outreach	N	Y	Y
Includes country FEs	N	N	Y

*** p < 0.01, ** p < 0.05, * p < 0.1 Robust standard errors shown in parentheses.

We also compare our findings with evidence from similar livelihoods programs implemented around the world. A recent study estimated the range of costs of economic inclusion programs implemented globally between $41 and $2,253 per beneficiary over an average of a little over 3 years of participation in 2011 PPP (Paul et al., 2021). Using the 2011 PPP and assuming a participation period of 3 years, our estimates suggest that the average cost of *Jeevika* participation was $235 if the beneficiary joined the program in 2008 (first phase) and $90 if the beneficiary joined the program in 2013 (second phase). Paul et al. (2021) also found that livelihoods and jobs programs implemented by governments had higher costs on average than those implemented by NGOs. In general, government-implemented programs tend to be more complex in operations, often layering multiple interventions and operating at a much larger and more diverse scale. Costs also varied because of different program objectives, design elements including intervention dosage or adequacy, sequencing and duration of interventions, and program context. Although not specifically identifying the impact of scale, the study of Paul et al. (2021) presents some evidence suggesting economies of scale of similar programs adopted across the world. For example, costs of the Sahel Adaptive Social protection program were higher in Burkina Faso ($260), where it targeted 18,000 households than in Mauritania ($570), where it targeted 2,000 households (Paul et al., 2021, World Bank, 2017).

Analyzing the National Rural Livelihoods Project (NRLP) implemented across India, Paul et al. (2021) found slightly lower costs of the NRLP compared to *Jeevika* in Bihar. A breakdown of cost components suggested that Bihar spent more on providing specialized funds and market links, and less on targeting costs. These differences likely reflect differences in scale and the gains from early implementation experience of *Jeevika* that may have brought down targeting costs. Comparing earlier formed and later formed SHGs across seven states, Kochar et al. (2020) suggest that early-stage SHGs in Bihar and Madhya Pradesh performed better in terms of access to resources, setup of federations, and overall pace of implementation, likely because Bihar and Madhya Pradesh already had significant experience with similar programs. In general, programs with an established delivery system have lower costs when identifying beneficiaries, constituting groups, and delivering repeated frontline services like savings facilitation and coaching (Paul et al., 2021). Many other state-level differences, including the scope of social inclusion, also are linked to differences in costs. For example, our analysis of the NRLM data indicates that Bihar has a high proportion of SHGs entirely composed of scheduled caste populations (23%) – reflecting both a higher representation and prioritization of marginalized social groups.

## 9 Discussion

This study brings together two seemingly disjoint streams of discourse in translating evidence from impact evaluations to policymakers – First, the past decade has seen an increasing emphasis, especially in international development, on incorporating cost estimates and cost-effectiveness and cost-benefit analyses into impact evaluations (Brown & Tanner, 2019). Second, while many development interventions often show positive impacts of pilot programs, pilot programs may fail to achieve similar success when scaled up (Vivalt, 2020, Bold et al., 2018). Yet, there is currently little to no discussion on how large-scale interventions may benefit from economies of scale resulting in lower costs, a factor that is especially crucial for group-based programs like SHGs, which are able to deliver services to many people at once (Dìaz-Martin et al., 2020).

Our analyses on economies of scale of SHG implementation in Bihar contribute to addressing this evidence gap through three key takeaways. First, we conducted a cost analysis to estimate the average costs of one of the largest SHG programs in the world over twelve years, showing per capita annual costs for each level of scale, and the extent to which the program can benefit from economies of scale. We found that annual per capita expenditure of *Jeevika* on basic program activities increased less than proportionately as scale increased – average annual program expenditure was $29 when the program had 100,000 members, $11-$13 when the program had one million members and was as low as $4-$6 with 10 million members. Comparing our findings with similar programs across the world, we found that average cost per beneficiary for *Jeevika* falls within the range of costs reported for economic inclusion programs funded by the World Bank. However, it remains important to note that information on program costs is very rarely reported in impact evaluations. For example, out of 97 impact evaluations of financial inclusion programs that are currently funded by the World Bank, only 20 studies reported information on costs, with only 15 studies collecting cost information at a disaggregated level (Paul et al., 2021).

Second, we found that the extent of economies of scale varies considerably with program activities. Our findings indicate that formation of higher-level federations and SHG-bank linkages can be achieved at a relatively low marginal cost. Evidence from other studies shows marked improvements in the performance of SHGs after the formation of VOs and CLFs. Specifically, Kochar et al. (2020) found that linking SHGs to VOs and CLFs is associated with improved financial access and use of funds, and higher expenditure and value of productive assets for households. Federations serve as platforms for creating women-led community institutions that can leverage community resources and perform negotiations for the collective economic upliftment of their SHGs and its members. From a policy perspective, our findings indicate that *Jeevika* can improve the cost-effectiveness of its programming by continuing to place a large emphasis on the formation of higher-level federations.

Third, we found that although the average high-cost loan amounts decreased by lower amounts in phase 2 compared to the pilot, the difference in program impacts did not translate to large differences in cost-effectiveness, after combining our cost estimates with impact estimates from the pilot phase and the scaled-up phase. The differences in impact fade away in the cost-effectiveness analysis because the lower implementation costs of the scaled-up model were able to fully offset the lower benefits. However, it is important to emphasize that cost savings from scale-up are not meaningful if the program fails to generate any impact after scaling up. While *Jeevika* did have positive effects on consumption and productive asset ownership for landless households after scaling up, the program had only modest average treatment effects on consumption asset ownership and no positive effects on productive asset ownership or women’s decision-making power (Hoffmann et al., 2021). Yet, because of cost savings from scaling up, *Jeevika* could have been equally cost-effective on these outcomes in the scaled-up phase if impacts on these outcomes were about half the impacts during the pilot.

The differences in costs, impacts, and cost-effectiveness between the pilot and the scale-up suggest a need for a more nuanced interpretation of our findings. Implementers and researchers must identify early on the factors that lead to better implementation quality and therefore higher impact. In the case of *Jeevika*, for example, implementation research found that the program was less consistent in applying some of the ritualized processes of group activities after its scale-up, and coordinators spent less time on gathering contextual information and adapting their messaging for mobilization (Hoffman et al., 2018, Majumdar et al., 2017). Along with these challenges, the program also lowered the value of the Initial Capitalization Fund (ICF) from INR 50,000 to INR 15,000. The ICF is provided to SHGs after they attain maturity in four to six months. SHG members can take turns to borrow out of the ICF pot for a variety of reasons, including consumption smoothing, debt reduction and productive investments. Reduction in ICF amounts may imply that groups were unable to take on a similar range of activities during the scale-up as during the pilot of the program. The importance of implementation research and particularly qualitative research is also highlighted by the finding that there were no large changes in the likelihood of bank linkage and linkage to federations after scale-up. This finding indicates that qualitative information about program implementation as in Majumdar et al. (2017) may be more meaningful when aiming to identify shifts in potential channels to produce meaningful impacts after scale-up.

From a policy perspective, these findings suggest that SHG programming at scale could increase its cost-effectiveness by prioritizing program elements that evaluations of pilot programs identify as critical for achieving positive impacts on women’s empowerment and asset ownership. Qualitative evidence indicates that gathering contextual information and adapting messaging for mobilization was critical for achieving positive effects on women’s empowerment. Strengthening these program elements at scale could increase the cost-effectiveness of *Jeevika* even with an increase in costs, as long as *Jeevika* maintains the elements that were critical for success during the pilot phase. Further, the costs of contextual information and mobilization are mostly one-time costs, which likely do not require long-term investments. Extrapolating the information presented in Table 5, we find that the project would need to generate less than half of the impact on women’s empowerment indicators in the scaled-up phase to remain as cost-effective as the pilot phase. Therefore, targeted investments in program components that were critical to achieve improvements in women’s empowerment during the pilot phase can likely retain the program’s cost-effectiveness at scale even when costs increase to twice the current amounts and impacts on intra-household-decision making power and asset ownership decrease after scale-up.

Minimizing tradeoffs between impacts and cost savings after scale-up also requires addressing factors that influence SHG dropout. Specifically, the leadership of higher-level federations plays an important role in conflict resolution (Reddy, 2012), likely because they comprise SHG members with skills that contribute to organizational capacity. Strengthening federations at scale can also facilitate an effective working relationship with Panchayati Raj Institutions, which could increase access to other public programs and entitlements. Further, strengthening VOs and CLFs at scale can likely generate effective linkages across government institutions and NGOs.

Studies have shown that implementing development interventions at scale is challenging. In a recent note on insights from research in development economies, Artuc et al. (2020) indicated that as programs grow, they are often met with political economy issues arising from low state capacity or poor bureaucratic management. In addition, it is likely that programs that generate positive effects are highly resource-intensive, and such resources are often not available to operate programs at scale. Indeed, the expansion of *Jeevika*’s SHG program faced similar challenges. Our study shows that it is critical to examine the costs and cost-effectiveness of development programs at different stages of program scale. Such analyses require longitudinal data on the program activities – specifically the components that are critical for program effectiveness, costs, and impact, and how these change with the number of program participants. These data are currently very scarce, including on SHG programs in other Indian states, which makes it challenging for decision makers to make evidence-based decisions about the scale-up of international development programs. For SHGs in particular it is critical to examine how changes in costs after scale-up are related to the group-based nature of SHGs. Recent studies indicate that training SHG members in a group setting could lead to more cost-effective outcomes than training SHG members individually (Grantham-McGregor et al., 2020). These differences in the cost-effectiveness of training of groups versus individuals may be even more pronounced after the scale-up of SHG programs. *Jeevika* data on specific cost categories did not include sufficient detail about group-based versus individual activities, but future studies should examine this question by collecting primary data on cost ingredients by economic activity, following existing guidelines and tools (Bhula et al., 2020, Siwach et al., 2019).

Regardless, however, our results show that the current emphasis on the reduced effectiveness after program scale-up is not sufficient and may result in a biased view about the possibility of achieving positive impacts at scale when studies fail to account for the existence of economies of scale. While program effectiveness may reduce after scale-up for most international development programs (Vivalt, 2020), the presence of economies of scale may justify the scaling of successful pilot programs even when their impact reduces with the number of program participants. Without such research, policy implications of the studies examining the relationship between impact and scale remain unclear. Importantly, however, examining the cost-effectiveness of programs is less meaningful if programs fail to produce positive impacts once scaled-up, highlighting the importance of implementation research to identify the factors and pathways to program impacts.

We recognize that the implementation and costs of large-scale SHG programs in India differs widely across contexts, showing the importance of studying women’s group implementation models (Desai et al., 2020). This study was able to triangulate findings on costs with previous research on the implementation and impact of SHGs in Bihar. However, the same in-depth research on implementation and costs is currently not available for most other Indian states. Future research could focus on the implementation and costs of SHG programming in other Indian states. Finally, the finding that *Jeevika* led to a reduction in interest rates charged by informal moneylenders (Hoffman et al., 2018, Kochar et al., 2020), may imply that as the program scales up further, general equilibrium effects will be realized over a much wider area, beyond the program villages. The average cost per beneficiary at scale will likely decrease further if the size of beneficiaries goes beyond the intended target participants. Future research should estimate both general equilibrium effects, and the program’s cost-effectiveness with respect to multiple outcome domains to better compare costs against outcomes that policymakers may value most.

## CRediT authorship contribution statement

**Garima Siwach:** Conceptualization, Formal analysis, Investigation, Methodology, Project administration, Writing – original draft, Writing – review & editing. **Sohini Paul:** Data curation, Investigation, Writing – original draft, Writing – review & editing. **Thomas de Hoop:** Conceptualization, Resources, Funding acquisition, Writing – original draft, Writing – review & editing.

## Declaration of Competing Interest

The authors declare that they have no known competing financial interests or personal relationships that could have appeared to influence the work reported in this paper.

## Footnotes

1 We could not estimate the same effects for the pilot phase because the pilot study does not report heterogenous effects for landless households.

2 We recognize that even in the presence of increasing returns to scale, marginal costs may increase while average costs continue decreasing, but this paper focuses on average costs to account for economies of scale in line with Tulloch et al. (2019).

3 Community Investment Funds are still considered a cost, but we do not hypothesize economies of scale for this cost category.

4 We conducted formal statistical testing for the presence of unit roots in time series using the Dickey-Fuller Test. The test rejected the presence of unit root (at p=0.05) after the series were log transformed for all variables, except for the formation of cluster-level federations.

5 Surendra (2020) reports that around half of Indian households are indebted, and that only 17% of those who borrowed in 2017 borrowed from an institutional source, demonstrating the importance of informal money lenders in the Indian context.

6 Because the two evaluations were conducted over four years apart, the definition of “high cost” varied across the two studies. The Phase 1 study defined high cost loans as loans with a monthly interest rate higher than 2%, while Phase 2 study considered loans with monthly interest rate higher than 4% as high cost loans.

7 We will present these benefit-cost ratios in a forthcoming study that includes longer-term impact estimates of the *Jeevika* program.

8 Specifically, in Uganda, there was a spike in operational costs between 2015 and 2016. BRAC’s annual reports suggest that this increase is likely due to additional expenses for the transformation of BRAC microfinance to a regulated credit institution (tier II) company (BRAC, n.d.).
